# Brincidofovir inhibits polyomavirus infection *in vivo*

**DOI:** 10.1128/mbio.01049-24

**Published:** 2024-07-02

**Authors:** Arrienne B. Butic, Zoe E. Katz, Ge Jin, Koji Fukushima, Masatoshi Hazama, Aron E. Lukacher, Matthew D. Lauver

**Affiliations:** 1 Department of Microbiology and Immunology, Penn State College of Medicine, Hershey, Pennsylvania, USA; 2 SymBio Pharmaceuticals Limited, Toranomon, Minato, Tokyo, Japan; The University of North Carolina at Chapel Hill, Chapel Hill, North Carolina, USA

**Keywords:** polyomavirus, antiviral agents, kidney, brain, brincidofovir

## Abstract

Polyomaviruses are species-specific DNA viruses that can cause disease in immunocompromised individuals. Despite their role as the causative agents for several diseases, there are no currently approved antivirals for treating polyomavirus infection. Brincidofovir (BCV) is an antiviral approved for the treatment of poxvirus infections and has shown activity against other double-stranded DNA viruses. In this study, we tested the efficacy of BCV against polyomavirus infection *in vitro* and *in vivo* using mouse polyomavirus (MuPyV). BCV inhibited virus production in primary mouse kidney cells and brain cortical cells. BCV treatment of cells transfected with MuPyV genomic DNA resulted in a reduction in virus levels, indicating that viral inhibition occurs post-entry. Although *in vitro* BCV treatment had a limited effect on viral DNA and RNA levels, drug treatment was associated with a reduction in viral protein, raising the possibility that BCV acts post-transcriptionally to inhibit MuPyV infection. In mice, BCV treatment was well tolerated, and prophylactic treatment resulted in a reduction in viral DNA levels and a potent suppression of infectious virus production in the kidney and brain. In mice with chronic polyomavirus infection, therapeutic administration of BCV decreased viremia and reduced infection in the kidney. These data demonstrate that BCV exerts antiviral activity against polyomavirus infection *in vivo*, supporting further investigation into the use of BCV to treat clinical polyomavirus infections.

**IMPORTANCE:**

Widespread in the human population and able to persist asymptomatically for the life of an individual, polyomavirus infections cause a significant disease burden in the immunocompromised. Individuals undergoing immune suppression, such as kidney transplant patients or those treated for autoimmune diseases, are particularly at high risk for polyomavirus-associated diseases. Because no antiviral agent exists for treating polyomavirus infections, management of polyomavirus-associated diseases typically involves reducing or discontinuing immunomodulatory therapy. This can be perilous due to the risk of transplant rejection and the potential development of adverse immune reactions. Thus, there is a pressing need for the development of antivirals targeting polyomaviruses. Here, we investigate the effects of brincidofovir, an FDA-approved antiviral, on polyomavirus infection *in vivo* using mouse polyomavirus. We show that the drug is well-tolerated in mice, reduces infectious viral titers, and limits viral pathology, indicating the potential of brincidofovir as an anti-polyomavirus therapeutic.

## INTRODUCTION

Found in a wide range of species, polyomaviruses are nonenveloped, double-stranded DNA viruses specific to their natural hosts (1). Polyomaviruses typically cause an asymptomatic and lifelong infection in immunocompetent individuals with periodic viral shedding. Under conditions of immune compromise, however, these viruses can resurge, leading to severe disease. In particular, two human polyomaviruses, BK polyomavirus (BKPyV) and JC polyomavirus (JCPyV), are the causative agents of several diseases in immunocompromised individuals (2–4). Both viruses are ubiquitous, with antibody seroprevalences in healthy populations reported to be greater than 95% and 60% for BKPyV and JCPyV, respectively (5, 6). Initial infection for both viruses is thought to occur through the respiratory or fecal-oral route (7, 8). The viruses then establish a lifelong persistent infection in multiple tissues, including the kidney, which is a major reservoir (9–13).

Kidney transplant patients represent a high-risk population for complications related to BKPyV infection. After transplantation, patients can exhibit asymptomatic viruria or viremia, which may precede the development of BKPyV-associated nephropathy (BKVAN) (14, 15). BKVAN affects up to 10% of kidney transplant patients and is a major cause of allograft dysfunction and failure (16–19). Reducing immunosuppression once elevated BKPyV viremia is detected is the mainstay of BKVAN management (20, 21). Such a therapeutic strategy carries the risk of allograft rejection and subsequent loss (22, 23).

Immunosuppression in JCPyV-infected individuals can lead to viral infiltration of the central nervous system, which precipitates a multitude of diseases, the dominant one being progressive multifocal leukoencephalopathy (PML). In PML, astrocytes and oligodendrocytes are infected by JCPyV, resulting in multifocal demyelination and a variety of deficits depending on lesion location, such as ataxia, cognitive loss, and behavioral changes (10). Prior to the development of highly active antiretroviral therapy (HAART), up to 8% of AIDS patients developed PML (24). Although the incidence of HIV-associated PML has declined with the implementation of HAART, the introduction of immunomodulatory drugs to treat inflammatory and autoimmune disorders, such as natalizumab for relapsing-remitting multiple sclerosis, has increased the incidence of drug-induced PML (25, 26). Management of PML typically involves discontinuing immunomodulatory therapies, but this intervention is often complicated by the development of immune reconstitution inflammatory syndrome, an inflammatory state that can aggravate neurological symptoms (27). Ultimately, there is a pressing clinical need for antiviral therapeutic strategies that can treat opportunistic polyomavirus infections like BKVAN and PML without the rebound inflammatory risks that accompany the restoration of immune activity.

BCV is a lipid-drug conjugate of cidofovir (CDV), a nucleotide analog, with demonstrated activity against a broad range of viruses (28, 29). BCV has been shown to reduce the replication of BKPyV in both primary human urothelial cells and renal proximal tubular epithelial cells (30, 31). Similarly, BCV treatment of JCPyV-infected immortalized human fetal brain cells reduced both viral DNA replication and genome copy numbers (32). BCV has also been found to decrease JCPyV replication and amounts of infectious progeny in human brain progenitor stem cell-derived astrocytes (33). The lipid moiety in BCV confers several advantages over CDV as an antiviral agent, such as increased cellular uptake, increased oral bioavailability, and reduced nephrotoxicity (28, 34). BCV is converted intracellularly into cidofovir diphosphate (CDV-PP), the active metabolite of CDV. The putative antiviral effects of CDV-PP result from its incorporation into replicating viral DNA, leading to inefficient DNA synthesis and an increased rate of chain termination. At higher concentrations, CDV-PP can directly inhibit virus-encoded polymerases (35).

Although BCV has been shown to inhibit BKPyV and JCPyV *in vitro* (31–33, 36), there is a lack of preclinical data on its efficacy against polyomavirus infections *in vivo*. In this study, we investigated the effects of BCV on polyomavirus infection using mouse polyomavirus (MuPyV), a natural murine pathogen. Like JCPyV and BKPyV in humans, MuPyV is a ubiquitous and silent pathogen in feral mice (37). MuPyV shares structural and genomic similarities with JCPyV and BKPyV and infects both the kidney and brain (38, 39). Immunocompromised mice infected with MuPyV exhibit increased viral replication and viremia (40). Utilizing MuPyV as a model, we demonstrate the efficacy of BCV both *in vitro* and *in vivo*, raising its potential as an anti-polyomavirus agent.

## RESULTS

### BCV inhibits MuPyV infection *in vitro*

**Fig 1:**
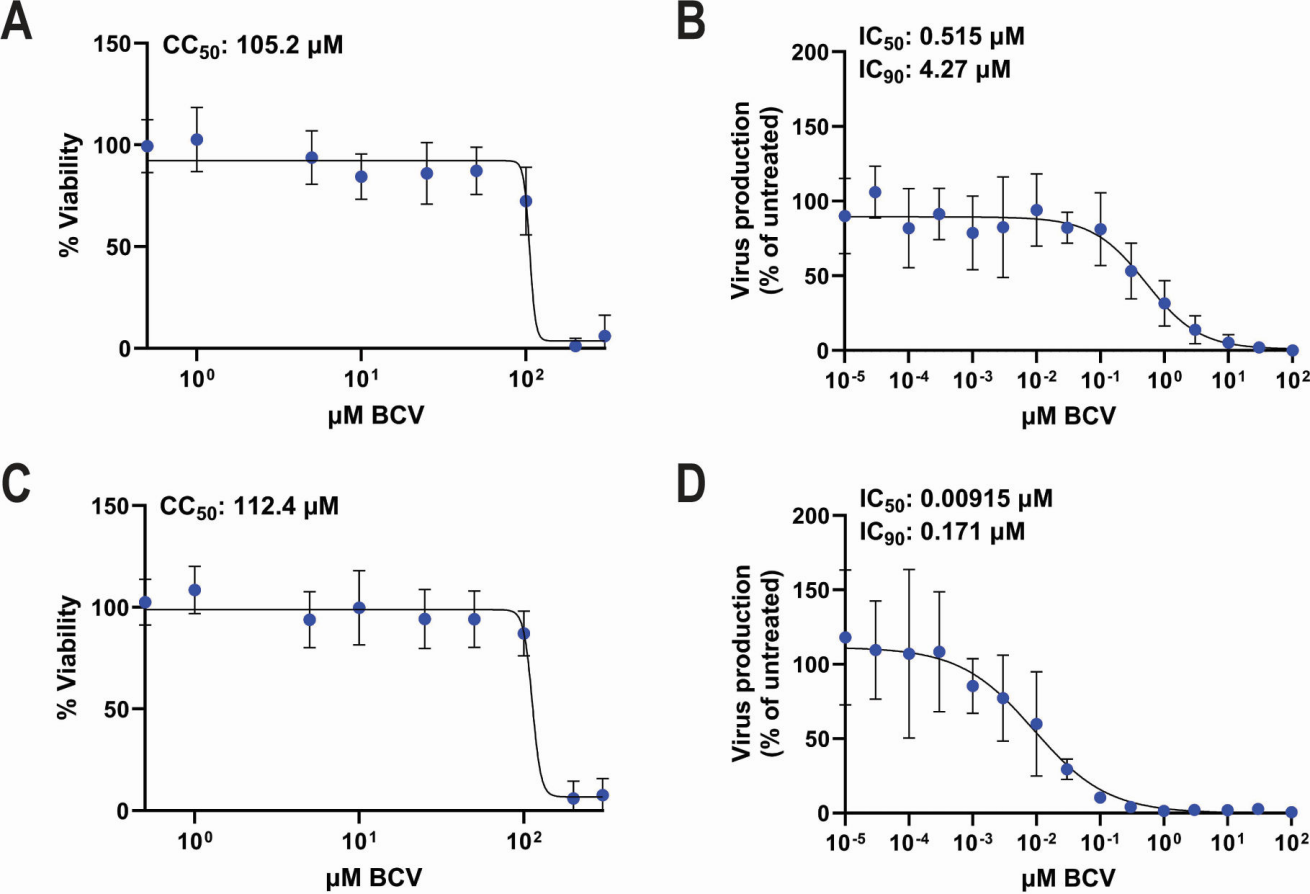
BCV inhibits MuPyV in primary renal and cortical cells. (**A**) Cell viability in AMK cells after 84 hours of BCV treatment. Data are from three independent experiments (*n* = 18). (**B**) Dose-response curve of viral inhibition in AMK cells. Cells were pretreated with BCV for 24 hours and then infected at a multiplicity of infection (MOI) of 0.1. Viral titers were measured at 60 hpi by plaque assay and normalized to viral titers in untreated cells. Data are from three independent experiments (*n* = 7). (**C**) Cell viability in primary cortical cells after 84 hours of BCV treatment. Data are from two independent experiments (*n* = 12). (**D**) Dose-response curve of viral inhibition in primary cortical cells. Cells were pretreated with BCV for 24 hours and then infected at an MOI of 0.1. Viral titers were measured at 60 hpi and normalized to viral titers in untreated cells. Data are from three independent experiments (*n* = 6).

To investigate the efficacy of BCV at inhibiting MuPyV, we first assessed the cytotoxicity of BCV in primary adult mouse kidney (AMK) epithelial cells. BCV was well-tolerated by the AMK cells, with a CC_50_ of 105 µM at 84 hours of treatment, which is the longest duration of BCV treatment used in this study (Fig. 1A). Given the tolerated range of BCV concentrations, we next assessed whether BCV displayed antiviral activity against MuPyV in AMK cells. Cells were treated with BCV for 24 hours prior to MuPyV infection, and infectious viral titers were measured at 60 hours post-infection (hpi) by plaque assay. BCV reduced infectious MuPyV production with an IC_50_ and IC_90_ of 0.515 and 4.27 µM, respectively (Fig. 1B). We next examined the cytotoxicity of BCV in primary mouse cortical cells, a mixed cell culture containing microglia, astrocytes, and oligodendrocyte precursor cells (41). As with the AMK cells, BCV was well-tolerated by the cortical cells with a CC_50_ of 112 µM (Fig. 1C). We then assessed the antiviral effect of BCV pretreatment in the cortical cells and determined an IC_50_ and IC_90_ of 9.15 nM and 0.171 µM, respectively (Fig. 1D). Together, these data show that BCV has anti-MuPyV activity significantly below its cytotoxic concentrations.

**Fig 2:**
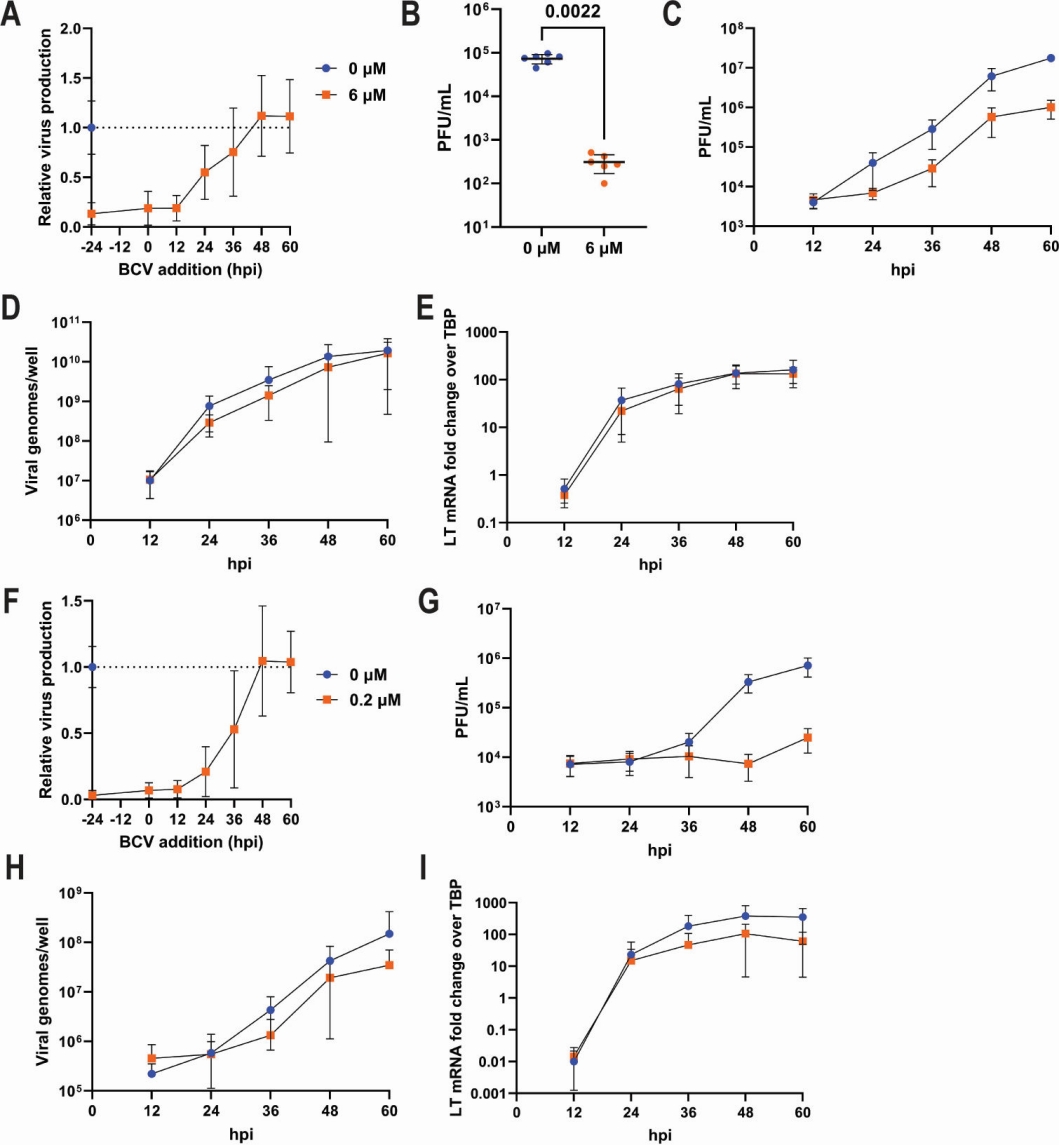
BCV inhibits MuPyV post-entry but does not inhibit viral DNA or RNA production. (**A**) Effect of BCV addition at different time points on virus production. AMK cells were infected at an MOI of 0.1, and BCV was added to the media at the indicated time points. Virus production was assessed at 60 hpi by plaque assay and normalized to untreated cells. Data are from five independent repeats (*n* = 15). (**B**) Effect of BCV on virus production after viral genome transfection. AMK cells were pretreated with BCV for 24 hours, transfected with viral DNA, and returned to BCV-containing media. Viral lysates were collected at 60 hours post-transfection and titered by plaque assay. Data are from two independent repeats (*n* = 6). (**C**) Effect of BCV on virus production throughout infection. AMK cells were pretreated with BCV, infected at an MOI of 0.1, and returned to BCV-containing media. Cell lysates were collected at the indicated time points, and virus production was assessed. Data are from two independent experiments (*n* = 6). (**D and E**) Effect of BCV on viral DNA and RNA production. AMK cells were treated as in panel **C**, and viral DNA (**D**) and RNA (**E**) were quantified at the indicated time points. Data are from two independent experiments (*n* = 6). (**F–I**) Effect of BCV on cortical cells. (**F**) Effect of BCV addition at different time points on virus production. Cortical cells were infected with an MOI of 0.1, and BCV was added to the media at the indicated time points. Virus production was assessed at 60 hpi by plaque assay and normalized to untreated cells. Data are from two independent repeats (*n* = 5). (**G**) Effect of BCV on virus production throughout infection. Cortical cells were pretreated with BCV, infected at an MOI of 0.1, and returned to BCV-containing media. Cell lysates were collected at the indicated time points and virus production was assessed. Data are from two independent experiments (*n* = 9). (**H and I**) Effect of BCV on viral DNA and RNA production. Cortical cells were treated as in panel **G**, and viral DNA (**H**) and RNA (**I**) were quantified at the indicated time points. Data are from two independent experiments (*n* = 7–9). Data were analyzed by the Mann-Whitney test (**B**).

The significant activity of BCV against MuPyV infection prompted us to define which step(s) of the virus lifecycle BCV affected. We first examined the effect of BCV addition at different time points relative to infection using a BCV concentration of 6 µM in AMK cells, which was slightly above the IC_90_ we observed. AMK cells treated with BCV up to 12 hpi showed a similar reduction in virus production at 60 hpi to that of cells pretreated for 24 hours, but the antiviral effect was largely absent in cells treated at 24 hpi or later (Fig. 2A). To rule out an effect of BCV on viral binding, entry, and uncoating, we next transfected BCV-treated AMK cells with viral DNA to bypass these steps. BCV treatment still resulted in a substantial reduction in virus production at 60 hpi, indicating that BCV inhibits MuPyV at a step after viral entry and genome uncoating (Fig. 2B). We then measured virus production over time out to 60 hpi, which was chosen as the endpoint to restrict virus replication to the initially infected cells. We found that BCV pretreatment inhibited the accumulation of infectious virus by 24 hpi, and this reduction was sustained out to 60 hpi (Fig. 2C). In contrast to the reduction in infectious virus, BCV treatment did not affect the levels of viral DNA or large T (LT) antigen mRNA over the same time period (Fig. 2D and E). In cortical cells, BCV treatment with IC_90_ concentration of 0.2 µM up to 12 hours post-infection substantially reduced infectious virus production at 60 hpi (Fig. 2F). BCV pretreatment prevented the accumulation of infectious virus, which was evident at 48 and 60 hpi (Fig. 2G). Despite a substantial effect on infectious virus production, BCV had a minimal effect on viral DNA and RNA accumulation (Fig. 2H and I). These data indicate that BCV inhibits MuPyV post-entry and uncoating, with minimal effect on viral DNA and RNA production.

**Fig 3:**
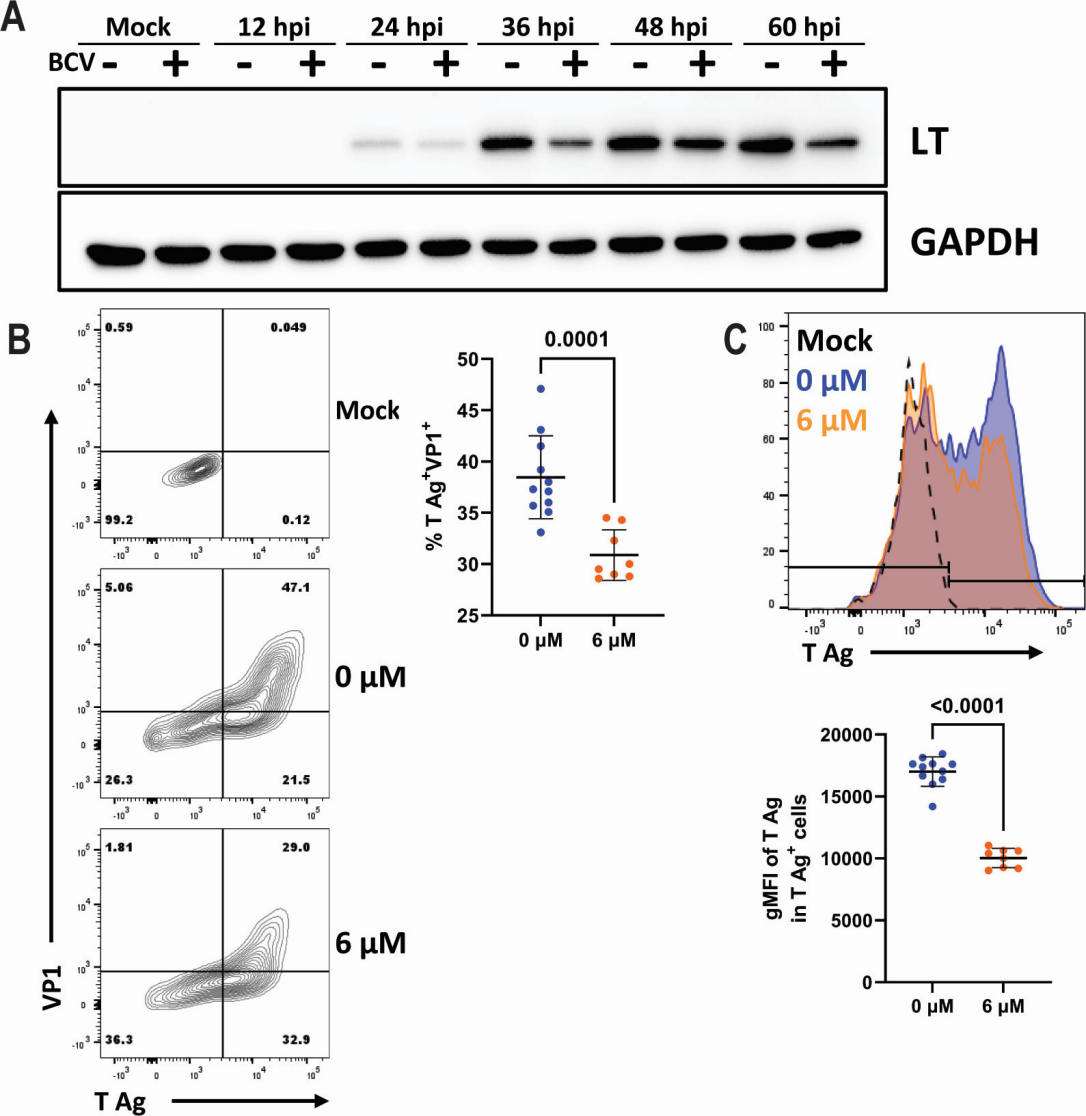
BCV reduces MuPyV large T antigen levels. (**A**) Western blot for LT antigen. AMK cells were pretreated with BCV, infected at an MOI of 0.1, and returned to BCV-containing media. Cell lysates were collected at the indicated time points, and protein levels were assessed. Data are representative of two independent experiments. (**B and C**) Analysis of T Ag and VP1 protein levels. AMK cells were pretreated with BCV, infected at an MOI of 0.1, and returned to BCV-containing media. Cells were trypsinized at 24 hpi and stained for T Ag and VP1. Protein expression was quantified by flow cytometry. (B, left) Representative plots of T Ag and VP1 expression in uninfected cells or infected cells treated with 0 or 6 µM of BCV. (B, right) Frequency of T ag^+^VP1^+^ cells. Data are from two independent experiments (*n* = 8–11). (C, top) Representative histogram of T Ag expression. (C, bottom) Levels of T Ag expression in the T ag^+^ cells. Data are from two independent experiments (*n* = 8–11). Data were analyzed by the Mann-Whitney test (**B and C**).

Due to the absence of an effect on viral DNA and RNA, we next examined whether BCV altered viral protein expression. Compared to untreated cells, AMK cells treated with BCV had reduced LT antigen by Western blot (Fig. 3A). We additionally examined viral protein expression by flow cytometry at 24 hpi. BCV treatment led to a reduction in the frequency of T antigen (T Ag)/VP1 double-positive cells (Fig. 3B). Within the T Ag-positive population, BCV treatment also caused a reduction in the level of T Ag expression (Fig. 3C). Together, these data indicate that BCV treatment leads to a reduction in MuPyV protein expression.

### Prophylactic treatment with BCV reduces acute MuPyV kidney and brain infection

**Fig 4:**
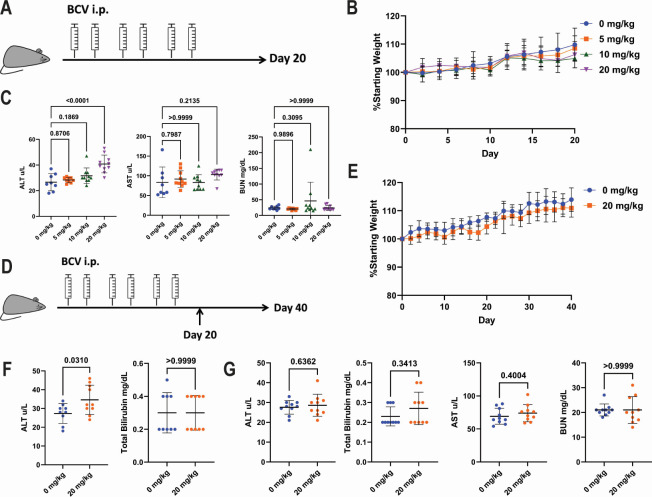
*In vivo* tolerability of BCV. (**A**) Schematic of BCV tolerability analysis. Mice received two doses of BCV a week for 3 weeks and were weighed every 2 days. After 20 days, mice were euthanized, and serum was collected for the analysis of ALT, AST, and BUN levels. (**B**) Weights of mice treated biweekly with the indicated doses of BCV for 3 weeks. (**C**) Serum ALT (left), AST (center), and BUN (right) levels in the mice after 3 weeks of BCV treatment. (**D**) Schematic of extended BCV tolerability analysis. Mice received BCV treatments as in panel **A** and were followed until 40 days after BCV initiation. Serum was collected at days 20 and 40. (**E**) Weights of mice treated biweekly with the indicated doses of BCV for 3 weeks. (**F**) Serum ALT (left) and total bilirubin (right) at day 20. (**G**) Serum ALT, total bilirubin, AST, and BUN at day 40. Data were analyzed by one-way ANOVA (**C**), unpaired *t* test: (F) ALT and (G) ALT/AST/BUN, or Mann-Whitney test: (F) bilirubin and (G) bilirubin. Data are from two independent experiments (*n* = 8–10).

The strong anti-MuPyV effects of BCV *in vitro* suggested that it could reduce virus infection *in vivo*. To examine this, we tested the tolerability of biweekly intraperitoneal (i.p.) administration of BCV (0, 5, 10, or 20 mg/kg of body weight/dose). Mice received 3 weeks of BCV treatment and were assessed for weight loss throughout treatment and for liver enzyme and blood urea nitrogen (BUN) levels at the conclusion of treatment (Fig. 4A). Mice treated with BCV doses up to 20 mg/kg of body weight (40 mg/kg of body weight/week) gained weight comparably to controls (Fig. 4B). Treatment with 20 mg BCV/kg of body weight caused a slight increase in serum alanine transaminase (ALT) but did not alter serum aspartate aminotransferase (AST) levels or BUN (Fig. 4C). Given the slight elevation in serum ALT levels with 20 mg BCV/kg of body weight, we next assessed whether this increase was transient and would resolve after the cessation of treatment. Mice were treated with vehicle or 20 mg BCV/kg of body weight as before but were followed for an additional 3 weeks after the termination of BCV administration (Fig. 4D). BCV-treated mice gained weight comparably to vehicle-treated mice for the duration of the study (Fig. 4E). Twenty days after the initiation of BCV treatment, serum ALT levels were again slightly elevated in the BCV-treated mice, but no increase was seen in total serum bilirubin, indicating the absence of severe liver injury or toxicity (Fig. 4F). At 40 days after the initiation of BCV and 24 days after the final BCV dose, no differences were observed in serum ALT, total bilirubin, AST, or BUN between vehicle- and BCV-treated mice, indicating the reversibility of the elevated ALT levels observed at day 20 (Fig. 4G). These findings match preclinical studies in humans, showing that BCV treatment is associated with moderate, transient elevations in serum ALT (42, 43). Together, these data indicate the tolerability of 3 weeks of BCV up to 20 mg/kg of body weight biweekly.

**Fig 5:**
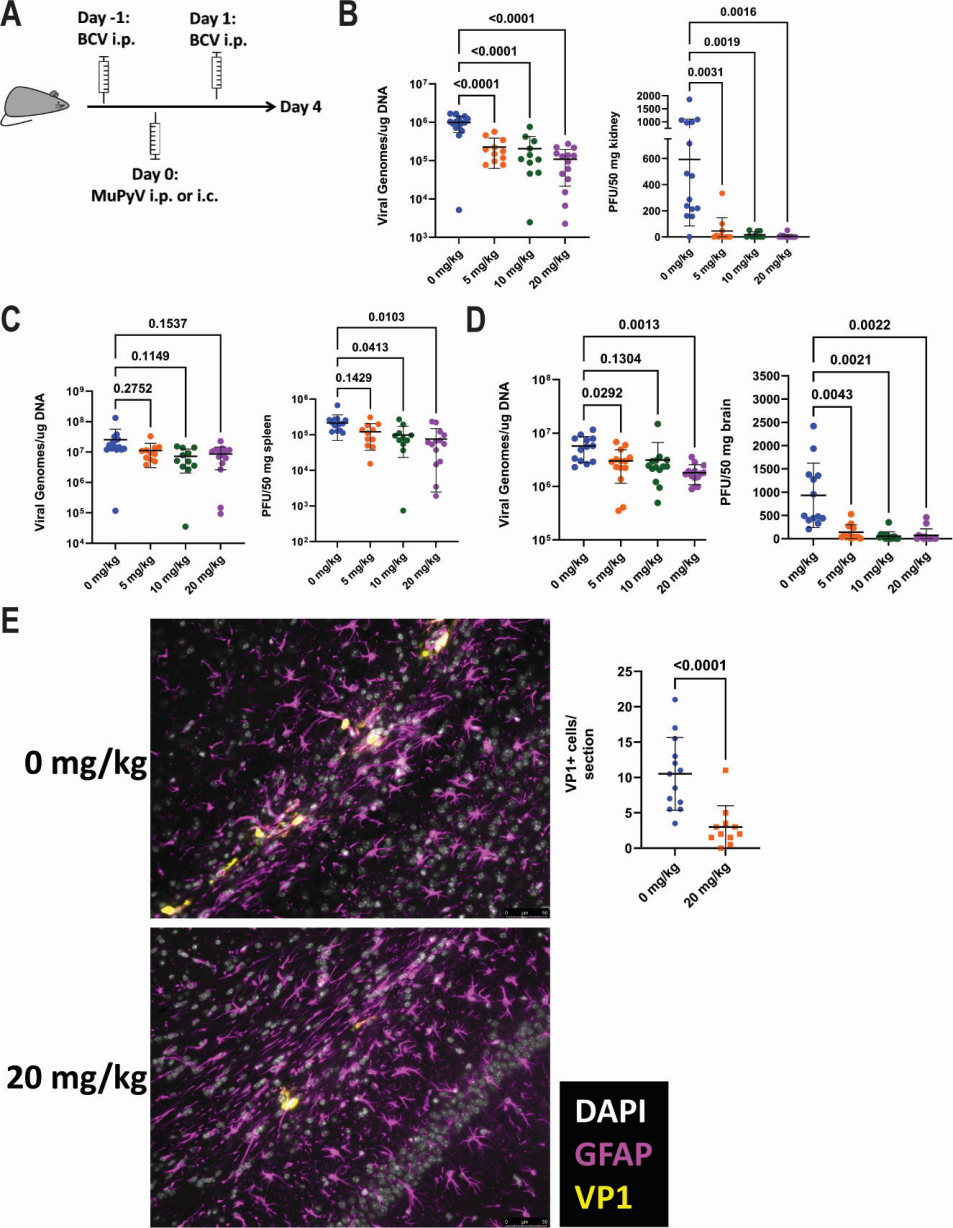
BCV treatment limits MuPyV kidney and brain infection. (**A**) Schematic of acute infection experiments with BCV treatment. Mice received BCV on days −1 and 1 and were infected with MuPyV either i.p. or i.c. on day 0. Mice were euthanized on day 4, and virus levels were assessed. Virus levels were quantified by qPCR for viral genomes or by plaque assay. (**B**) Kidney virus levels in i.p.-infected mice. Data are from four independent experiments (*n* = 10–15). (**C**) Spleen virus levels in i.p.-infected mice. Data are from four independent experiments (*n* = 11–15). (**D**) Brain virus levels in i.c.-infected mice. Data are from three independent experiments (*n* = 13–14). (**E**) GFAP and VP1 immunofluorescence staining of brain sections from vehicle- or 20 mg/kg BCV-treated mice. Left: representative images of brain regions with infected VP1^+^ cells. Right: quantification of the frequency of VP1^+^ cells. Data shown are the average number of VP1^+^ cells in two sections from different brain regions per mouse. Data are from two independent experiments (*n* = 11–13). Data were analyzed by Brown-Forsythe and Welch ANOVA tests (B–D) or Mann-Whitney test (**E**).

We next asked whether BCV treatment could limit acute MuPyV infection in the kidney and brain. Mice were administered a dose of BCV the day before and a dose the day after MuPyV infection (Fig. 5A). Mice were infected i.p. to assess the inhibition of kidney infection, whereas for the inhibition of brain infection, mice were infected intracranially (i.c.). In mice infected i.p., all BCV doses caused a significant decrease in viral DNA and plaque-forming units (PFU) in the kidney compared to vehicle-treated mice (Fig. 5B). Although BCV treatment did not cause a significant decrease in viral DNA in the spleens of these mice, doses of 10 and 20 mg/kg of body weight did result in a significant reduction in viral PFU in the spleen (Fig. 5C). In mice infected i.c., BCV treatment resulted in a reduction in both viral DNA and PFU in the brain (Fig. 5D). To further examine the effect of BCV on MuPyV brain infection, we stained brain sections from vehicle- or 20 mg/kg BCV-treated mice for VP1 and glial fibrillary acidic protein (GFAP), a marker for astrocytes (44). In comparison to vehicle-treated mice, BCV-treated mice had a reduced frequency of VP1^+^ cells (Fig. 5E). These data demonstrate that BCV has *in vivo* antiviral activity against polyomavirus infections in both the kidney and the brain.

### BCV inhibits chronic MuPyV kidney infection

**Fig 6:**
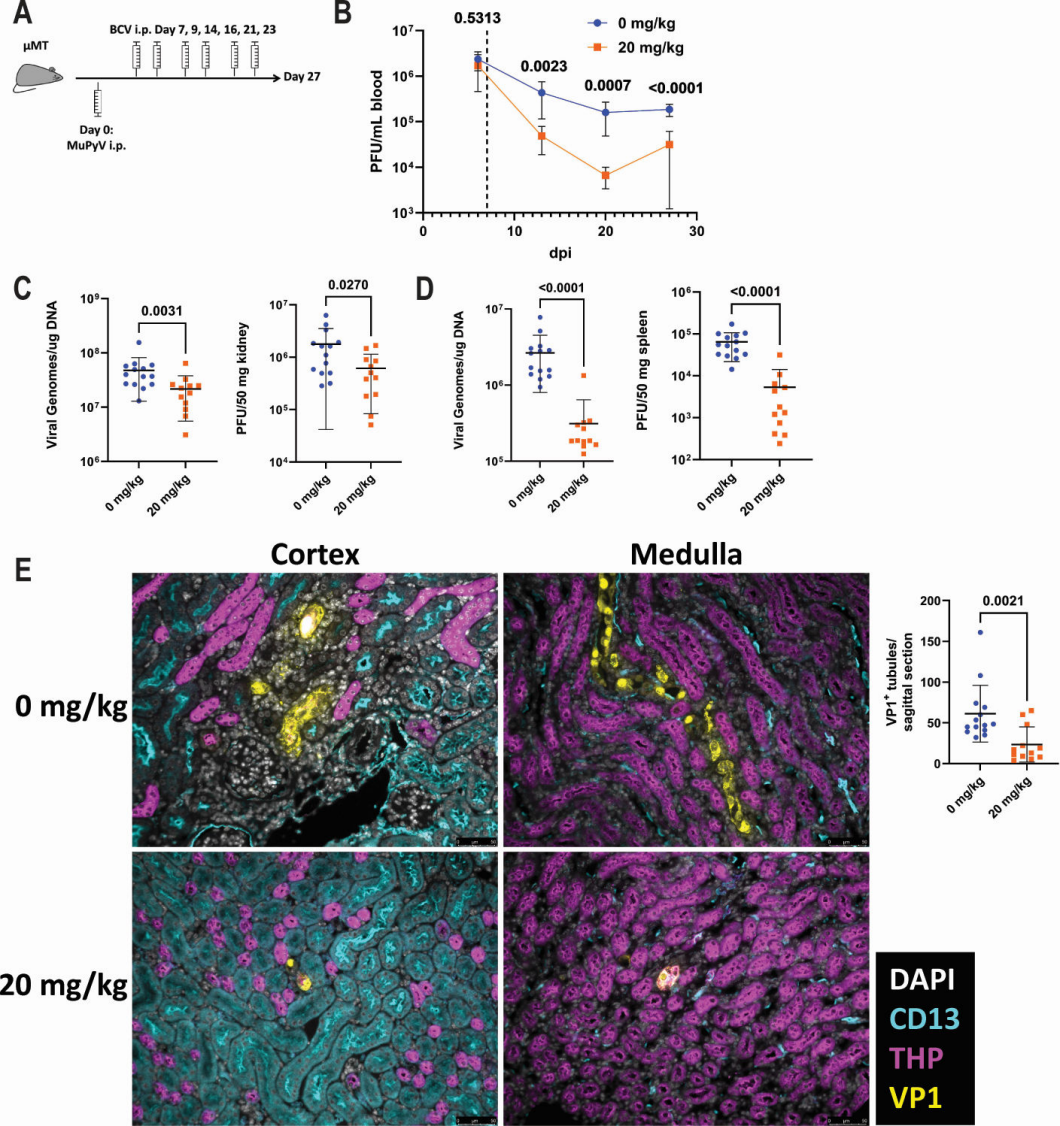
BCV reduces viral burden and limits kidney pathology during chronic infection. (A) µMT mice were infected with MuPyV, and viral load in the blood was measured weekly by plaque assay. Starting at 7 dpi, mice received biweekly injections of 0 or 20 mg BCV/kg of body weight. At 27 dpi, mice were euthanized, and virus levels in the spleen and kidney were quantified. (B) Blood was collected weekly, and virus titers were quantified by plaque assay. The dashed line indicates the start of BCV administration. Data are from three independent experiments (*n* = 12–14). (C) Viral load in the kidney quantified by qPCR for viral DNA (left) or plaque assay (right). Data are from three independent experiments (*n* = 12–14). (D) Viral load in the spleen quantified by qPCR (left) or plaque assay (right). Data are from three independent experiments (*n* = 12–14). (E) Sites of virus infection in the kidneys of vehicle- or BCV-treated µMT mice. Left: sites of virus infection in the kidney identified by VP1 immunofluorescence. Right: quantification of the number of VP1^+^ tubules per kidney section. Data are from three independent experiments (*n* = 12–14). Data were analyzed by two-way ANOVA (B) or Mann-Whitney test (C–E).

The efficacy of prophylactic BCV administration at limiting MuPyV kidney infection led us to ask whether BCV could be given therapeutically to treat an existing kidney infection. To test BCV treatment of an established kidney infection, we infected µMT mice, which lack mature B cells and develop a chronic MuPyV infection. MuPyV-infected µMT mice displayed chronic viremia, with infectious virus readily detected in the blood by plaque assay, and developed PyV-driven kidney pathology by 4 weeks post-infection (40). µMT mice were infected and began receiving biweekly vehicle or 20 mg BCV/kg of body weight for 3 weeks starting 7 days post-infection (dpi) (Fig. 6A). Blood was collected weekly to track viremia, and virus levels in the kidney and spleen were quantified at 27 dpi. Mice displayed significant viremia by 6 dpi, prior to the start of BCV treatment. Following the initiation of treatment, mice receiving BCV showed a significant reduction in viremia compared to vehicle-treated mice (Fig. 6B). The kidneys of the µMT mice contained titers of infectious MuPyV that were 1,000–10,000× higher than what was observed in the kidneys of the acutely infected wild-type mice. Despite the elevated virus levels, BCV treatment still led to a reduction in both viral PFU and viral DNA (Fig. 6C). BCV also reduced both viral PFU and viral DNA in the spleen (Fig. 6D). We also examined virus infection in the kidney by immunofluorescence. Kidney sections were stained for VP1 to identify the sites of virus infection, as well as for CD13 and Tamm-Horsfall Protein (THP), which label proximal tubules and distal tubules/ascending limb of the Loop of Henle, respectively (45, 46). Consistent with the reduction in virus levels, BCV-treated mice had a significant reduction in the number of VP1^+^ tubules (Fig. 6E). These data demonstrate that therapeutic treatment with BCV during chronic MuPyV kidney infection can reduce viral burden and virus pathology within the kidney.

## DISCUSSION

No drug is currently approved for the treatment of diseases caused by human polyomaviruses. Our work provides evidence that BCV shows *in vitro* and *in vivo* efficacy against MuPyV, a ubiquitous member of the virome of feral mice. BCV reduces infectious MuPyV titers in primary murine kidney and brain cortical cells at non-cytotoxic concentrations. Mice tolerate BCV at doses up to 20 mg/kg of body weight with no weight loss and modest, reversible changes in liver damage markers. In acutely infected mice, BCV reduces viral loads in the kidney, spleen, and brain. Importantly, BCV administration post-infection lowers infectious viral titers and viral DNA in chronically infected, immunodeficient mice.

Previous work supports the use of BCV against polyomavirus infections *in vitro*. BCV was shown to inhibit BKPyV replication in human fetal lung fibroblast cells at an EC_50_ of 0.13 µM (36). In BKPyV-infected primary human urothelial cells, BCV demonstrated an EC_50_ of 0.27 µM (31). The EC_50_ of BCV for JCPyV infections *in vitro* is reported to be 45 and 5.55 nM for immortalized human fetal brain cells and primary human brain progenitor-derived astrocytes, respectively (32, 33). In our system, BCV displayed an IC_50_ of 0.515 µM for primary AMK cells and an IC_50_ of 9.15 nM for primary mouse brain cortical cells (Fig. 1B and D). AMK cells grow to a higher cell density and produce 10 times higher MuPyV titers than cortical cells (Fig. 2C and G), which can result in the difference in IC_50_ for BCV between these primary cells (31).

BCV is a lipid conjugate prodrug of CDV, an acyclic nucleoside phosphonate that is converted into its active metabolite, CDV-PP (47, 48). CDV-PP acts as an alternative substrate for DNA polymerase, replacing deoxycytidine triphosphate, and is thus incorporated into growing DNA strands, impairing DNA extension and resulting in chain termination (35, 49, 50). Based on this, the antiviral effect of BCV treatment is expected to result in a reduction in viral DNA and RNA. However, we observed minimal change in viral DNA or RNA in either cortical or AMK cells by 60 hpi, an endpoint within a single replication cycle in initially infected cells (Fig. 2D, E, H, and I). Although other studies have shown a reduction in viral DNA or RNA levels, the duration prior to when this decline was seen extends beyond a single viral replication cycle. For example, in human fetal brain cells infected with JCPyV, BCV administration resulted in lower JCPyV DNA copy numbers at 4 dpi (32). Another study showed a significant reduction in JCPyV DNA with BCV treatment by 7 dpi, but viral DNA levels were not drastically altered at earlier time points (33). A BCV-associated drop in viral DNA levels *in vitro* may partly be the result of limiting virus production and subsequent spread and replication in new cells.

Although BCV treatment did not significantly reduce viral DNA or RNA in either mouse kidney or brain cortical cells (Fig. 2D, E, H, and I), drug treatment reduced the expression of T Ag protein (Fig. 3). BCV treatment of cells transfected with viral DNA resulted in lower viral titers, indicating that BCV does not affect early stages prior to viral DNA replication (Fig. 2B). These data suggest that BCV inhibits MuPyV by impairing viral protein expression without affecting viral genome replication or transcription. A possible explanation of how a nucleoside analog may reduce viral protein levels, but not viral DNA or RNA levels, is translation repression resulting from DNA damage. Double-stranded DNA breaks repress translation through ubiquitination and degradation of core ribosomal proteins (51–53). CDV treatment in cells induces DNA damage, which is exacerbated in transformed and proliferating cells; PyV T antigens drive infected cells into the S phase (54–56). Concomitant with this, PyV infection activates and usurps the DNA damage response (DDR) pathways for genome replication (57–59). A synergistic effect of BCV treatment in the presence of PyV infection could exacerbate this DDR or activate additional response pathways, triggering translation inhibition as infection progresses. Having evolved to co-opt the DDR, the virus can still undergo DNA replication but does not have a mechanism for overcoming translation suppression. This is consistent with our finding that BCV reduced T Ag^+^VP1^+^ cells, which are in the late stage of infection (Fig. 3B).

One factor that may differentially influence how BCV works against MuPyV is its absence of a viral DNA polymerase. Many of the viruses against which BCV is effective, such as vaccinia virus, adenovirus, and HCMV, encode their own DNA polymerase (60–62). In contrast, polyomaviruses rely on host cellular polymerases and their LT Ag, which is critical for the initiation of DNA replication and recruitment of cellular proteins such as DNA primase and polymerase (63). Active forms of other acyclic nucleoside phosphonates exhibit a higher affinity for viral polymerases than cellular polymerases, denoting that CDV-PP may have a poor affinity as a substrate for mammalian polymerases (48, 64). This is supported by the finding that, in a cell-free system utilizing purified proteins, CDV-PP was found to marginally inhibit DNA replication of SV40, BKPyV, and JCPyV (65).

BCV reduces MuPyV DNA and infectious viral titers during both acute and chronic infection (Fig. 5B through D and 6B through D). This reduction in DNA is consistent with *in vitro* data indicating that BCV reduced infectious virus production because the viral DNA measured *in vivo* represents multiple cycles of infection and replication. A reduction in viral DNA is expected to occur after multiple rounds of infection have occurred, as inhibition of infectious virus production leads to reduced infection of new cells and, therefore, reduced subsequent viral DNA replication. Mice acutely infected with MuPyV and prophylactically treated with BCV demonstrate a decrease in kidney viral DNA and infectious virus at doses as low as 5 mg/kg of body weight given twice a week (Fig. 5B). The reduction in spleen viral DNA and infectious virus is less significant, possibly due to the spleen having 10-fold higher levels of viral DNA than the kidney (Fig. 5B and C). Mice infected i.c. with MuPyV also exhibit a decrease in brain viral DNA and infectious virus at a dose as low as 5 mg/kg of body weight, with the most substantial decrease seen at 20 mg/kg of body weight (Fig. 5D). A prior study employing a dose of 20 mg BCV/kg of body weight found that this was sufficient to protect 100% of mice from lethal vaccinia virus infection (66).

Diseases caused by polyomavirus infections, such as BKVAN and PML, typically occur in immunocompromised humans. As early detection of these conditions is difficult, the window for therapeutic intervention often occurs when the individual already has elevated virus levels. In this study, we administered BCV to immunocompromised mice starting at 7 dpi, by which point the mice had developed substantial viremia (Fig. 6A and B). BCV treatment of these mice resulted in dramatically lower viral DNA and infectious titers in both the kidney and the spleen as well as reduced infectious titers in the blood (Fig. 6B through D). This provides compelling preclinical evidence that BCV given during an established polyomavirus infection can lower viral loads in the kidney and systemically. A reduction in viral infection would be expected to lower virus-associated inflammation and viral antigen levels, which may improve antiviral T-cell responses. CD8 T cells in PML patients express the inhibitory programmed cell death protein 1 (PD-1) receptor, and inflammation may upregulate its ligand, programmed death ligand 1 (PD-L1), on infected cells. Of note, MuPyV-specific brain-infiltrating T cells also upregulate PD-1, and PD-L1 is expressed by glial cells in i.c. inoculated mice (67, 68). By reducing infection, BCV may enhance T-cell immunity to polyomavirus infection, possibly via reduced inhibitory receptor/ligand interactions, and thereby further promote viral control and restore polyomavirus to its original station as a silent, persistent infection.

Although clinical studies have reported no benefit to the use of CDV in PML patients (69–71), clinical reports support the use of BCV against polyomavirus infections. This is likely due to the advantages that BCV has over CDV, namely increased oral bioavailability and higher cellular uptake facilitated by BCV’s lipid moiety (28, 72). In a pediatric kidney transplant patient with BKVAN, BCV reduced BKPyV viral loads in the plasma and urine, and serum creatinine levels, indicative of kidney function, returned to normal levels (73). In a later open-label and expanded access study, BCV was administered to an adult patient with BKVAN, prompting a decrease in BKPyV plasma viral loads (74). Although kidney function had deteriorated prior to BCV administration, function was retained post-administration. Lower BKPyV loads were also reported in the case of a stem cell transplant patient treated with BCV after developing a mixed infection with multiple viruses, including BKPyV (75). JCPyV viral loads in the serum were also reduced after BCV administration in a patient with PML whose neurological symptoms stabilized (76). In another study, four patients diagnosed with natalizumab-associated PML were treated with BCV and mirtazapine, an antidepressant that may counter JCPyV infection by antagonizing the virus’s 5-HT_2_ entry receptor (77–79). All patients displayed a reduction in viral copies in cerebrospinal fluid, and three exhibited improved neurological function. In several of these studies, BCV treatment reduced PyV burden in the blood or CSF by approximately 10-fold over the course of several months, whereas our treatment of chronic MuPyV infection led to a 10-fold reduction in viremia within several weeks (Fig. 6B). The model of chronic MuPyV infection used in this study has higher virus levels than a previously developed model of MuPyV-associated nephropathy following kidney transplant (80). The BCV-mediated reductions in virus levels seen in this chronic infection model further support the efficacy of BCV in controlling PyV infections.

Taken altogether, we demonstrate that BCV exhibits anti-polyomavirus activity in both cell culture and a pre-clinical animal model. Using primary mouse kidney and brain cells, we show that BCV is well-tolerated *in vitro*, with CC_50_ values above 100 µM and IC_50_ values > 2 orders of magnitude below the CC_50_. Our work suggests that BCV impedes polyomavirus production through the disruption of viral protein expression without affecting viral DNA or RNA. We also demonstrate that C57BL/6 mice tolerate biweekly administration of BCV at concentrations up to 20 mg/kg of body weight with no weight loss and minimal, reversible alterations in liver damage markers. Importantly, BCV systemically ameliorated both acute and chronic polyomavirus infections *in vivo*. BCV treatment reduced viral DNA and infectious viral titers in the brains and kidneys of acutely infected mice. Chronically infected immunodeficient mice displayed a decline in viremia as well as a reduction in viral DNA and infectious viral titers in the kidney and spleen after BCV treatment. In summary, our study highlights BCV as a candidate antiviral agent against human polyomaviruses in both prophylactic and therapeutic settings.

## MATERIALS AND METHODS

### Brincidofovir

The BCV used in this study was generously provided by Symbio Pharmaceuticals Limited as a 10 mg/mL stock solution.

### Mice and infections

C57BL/6 and µMT mice were purchased from Jackson Laboratories and housed and bred under specific pathogen-free conditions, maintained through routine testing of adult sentinel mice for common murine pathogens, in a designated barrier facility. Mice aged 6–12 weeks were used for experiments. For BCV administration, mice were injected i.p. with BCV diluted in PBS. For experiments examining kidney and spleen infection, mice were injected i.p. with 1 × 10^6^ PFU of MuPyV. For brain infection, mice were anesthetized and injected i.c. with 3 × 10^5^ PFU of MuPyV. Serum chemistry measurements were performed by the Department of Comparative Medicine at the Penn State College of Medicine.

### Isolation of primary mouse cells

Adult mouse kidney epithelial cells were isolated as previously described (81). Mixed cortical cells were isolated based on a protocol for isolating and culturing murine astrocytes (41). Brains were removed from 4-day-old C57BL/6 pups, and cortices were dissected out after the removal of meninges. Cortices were minced, digested using 2.5% trypsin in Hanks’ Balanced Salt Solution, dissociated into a single-cell suspension, and then plated in high-glucose Dulbecco’s modified Eagle medium (DMEM) with 10% fetal bovine serum and penicillin/streptomycin and ciprofloxacin.

### Virus infections *in vitro*

All experiments were performed using the A2 strain of MuPyV. AMK and primary murine cortical cells were seeded at 1.5 × 10^5^ cells/well. The following day, media were replaced with media containing BCV. After 24 hours of drug treatment, cells were washed with Iscove’s modified DMEM with 0.1% bovine serum albumin prior to infection with the indicated MOI. Infections were carried out at 4°C for 1.5 hours; the cells were then washed and returned to culture media containing BCV. For viral genome transfection, two plasmids carrying halves of the viral genome were digested and ligated together and then transfected in AMK cells pretreated with BCV. For the quantification of infectious virus production, the cells and supernatant were collected, subjected to three rounds of freeze/thaw, pH adjusted to 8–8.5, and centrifuged to remove cell debris. Plaque assays were performed in A31 fibroblasts as previously described (81). Viral titers were calculated as PFU/mL or normalized by experiment to BCV-untreated samples.

### Quantification of viral DNA and RNA

DNA and RNA were isolated from primary cells using TRIzol Reagent (Thermo Fisher Scientific), and DNA was isolated from tissues using the Wizard Genomic DNA Purification Kit (Promega). Viral RNA levels were quantified by TaqMan qPCR and normalized to TATA-box binding protein (82). Viral DNA was quantified by TaqMan qPCR and compared to a standard curve (83).

### T antigen quantification by flow cytometry

AMK cells were treated for 24 hours with BCV prior to infection with MuPyV at an MOI of 1. Following infection, the cells were returned to BCV-containing media. At 24 hpi, cells were trypsinized and treated with eBioscience Fixation/Permeabilization reagent (ThermoFisher Scientific). The cells were stained with rat anti-T Ag (1:1,000) and rabbit anti-VP1 (1:1,000) followed by staining with anti-rat and anti-rabbit secondaries (1:200). Samples were processed on a BD Symphony flow cytometer, and data were analyzed in FlowJo.

### Western blots

Whole-cell lysates were prepared with RIPA Lysis Buffer System supplemented with protease inhibitors (Santa Cruz Biotechnology), and protein was quantified by the Pierce BCA Protein Assay Kit (ThermoFisher Scientific). A total of 15 µg of boiled protein with SDS loading dye containing 2-betamercaptoethanol was run on 10% SDS-PAGE gels. Following the transfer, polyvinylidene difluoride membranes were blocked in 5% blocking buffer prepared using non-fat dry milk dissolved in Tris-buffered saline with 0.1% Tween-20 (TBST). Membranes were incubated overnight at 4°C or for 2 hours at room temperature with rat anti-T antigen or rabbit anti-GAPDH (Cell Signaling Technology) in 1% milk in TBST. Blots were washed with TBST followed by a 3-hour incubation with horseradish peroxidase conjugated secondary antibodies purchased from BioLegend in 1% milk in TBST. Blots were washed with TBST and developed with SuperSignal West Pico PLUS Chemiluminescence Substrate (ThermoFisher Scientific).

### Quantification of cell viability

AMK and murine cortical cells were seeded at 5 × 10^3^ cells/well in 96-well plates. The following day, the media were replaced with BCV-containing media, and the cells were cultured for 84 hours. Cells were treated for 2 hours with PrestoBlue Cell Viability Reagent (ThermoFisher Scientific), and the absorbances at 570 and 600 nm were measured. Cell viability was determined by normalization to samples without BCV treatment.

### Immunofluorescence staining

Kidneys were removed from mice and fixed overnight in neutral buffered formalin (NBF). For brain fixation, mice were perfused with NBF, and whole heads were fixed overnight in NBF prior to brain removal. Tissues were then paraffin-embedded and sectioned. Kidney and brain sections were processed and stained as previously described (84). Sections were stained with goat anti-mouse GFAP (Abcam), goat anti-mouse CD13 (Abcam), rat anti-mouse THP (R&D), and rabbit anti-VP1 antibodies. Secondary antibodies used were anti-goat Alexa Fluor 488 (Jackson Immunoresearch), anti-rabbit Alexa Fluor 555 (Abcam), and anti-rat Alexa Fluor 647 (Jackson Immunoresearch). Slides were mounted with Prolong Gold Antifade Mountant with DAPI (ThermoFisher Scientific). Images were acquired with a Leica DM4000 fluorescence microscope.

### Statistical analysis

Statistical analyses were performed using Prism (GraphPad). Exact *P* values are displayed in the figures, and *P* values of ≤ 0.05 were considered significant. Sample sizes are indicated in the figure legends and represent individual mice or biological replicates. The statistical tests used are listed in the figure legends. The frequency of VP1^+^ cells and tubules was quantified in a blinded fashion; no blinding was employed for other experiments. All data presented in the graphs are displayed as the mean ± SD.

## Data Availability

No data sets were generated in this study. All data supporting the findings of this study are presented in this paper.
